# The effect of different prebiotics on intestinal probiotics in newly diagnosed diabetic patients

**DOI:** 10.1002/fsn3.3709

**Published:** 2023-09-24

**Authors:** Yu Zhang, Lidan Yang, Yitian Wu, He He, Yuping Zeng, Zhenmei An, Weiguo Jia

**Affiliations:** ^1^ Department of Endocrinology and Metabolism, West China Hospital Sichuan University Chengdu Sichuan China; ^2^ Department of Laboratory Medicine, West China Hospital Sichuan University Chengdu Sichuan China; ^3^ Department of Medical Genetics, Key Laboratory of Birth Defects and Related Diseases of Women and Children of MOE, West China Second Hospital Sichuan University Chengdu Sichuan China; ^4^ The Center of Gerontology and Geriatrics, National Clinical Research Center of Geriatrics, West China Hospital Sichuan University Chengdu Sichuan China

**Keywords:** degradation rate, diabetes, intestinal flora, prebiotics, probiotics

## Abstract

Prebiotics exert favorable effects on the host through interactions with probiotics, and their beneficial impacts have been extensively validated across various chronic ailments, including diabetes. This study presents findings from a case–control investigation involving 10 individuals with type 2 diabetes mellitus (T2DM) and 10 healthy counterparts. Fresh stool specimens were collected from all participants. Following a 24‐h fermentation period in mediums containing xylitol and mannitol, the observed increase in *Lactobacillus* abundance within the case group exceeded that of the control group. Similarly, in mediums containing soluble starch, choline, and L‐carnitine, the augmentation of *Bifidobacterium* within the case group surpassed that of the controls. Notably, a statistically significant divergence in sugar degradation rate emerged between the case and control groups, specifically in the medium harboring lactulose and isomalto‐oligosaccharides. Remarkably, the degradation rate of lactulose exhibited a positive correlation with the expansion of *Bifidobacterium* (*R* ^2^ = .147, *p* = .037). Likewise, the degradation rate of isomalto‐oligosaccharides demonstrated a positive correlation with *Bifidobacterium* proliferation (*R* ^2^ = .165, *p* = .041). In conclusion, prebiotics like xylitol and mannitol exhibit the capacity to enhance intestinal probiotic populations in individuals newly diagnosed with diabetes. The modifications in the intestinal flora homeostasis of diabetic patients may be evidenced by alterations in the degradation rate of specific prebiotic substrates.

## Graphical abstract

In this work, Prebiotics such as xylitol and mannitol can increase intestinal probiotics in newly diagnosed diabetic patients, which may have a certain inhibiting effect on the occurrence and development of diabetes. The changes in the homeostasis of the intestinal flora of diabetic patients may be manifested by the degradation rate of certain types of prebiotics. Adjusting the intestinal flora through prebiotics may become a new method of DM treatment.

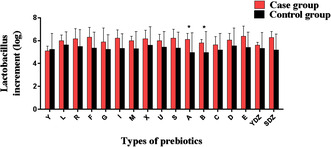

## 1 INTRODUCTION

In recent decades, the incidence and prevalence of type 2 diabetes mellitus (T2DM) has been dramatically increased, contributing great burden to global health economies (Chatterjee et al., 2017; Cole & Florez, 2020; Holman et al., 2015; Salgaço et al., 2019). At present, academic studies believe that the main causes of T2DM include not only genetic factors, but also obesity, a sedentary lifestyle, and a high‐sugar and high‐fat diet (Chatterjee et al., 2017). However, many studies in recent years have confirmed that cardiovascular diseases, autoimmune diseases, obesity, and T2DM are closely related to intestinal flora (Roager et al., 2019). The occurrence and development of T2DM may be related to the composition of the intestinal flora and their metabolites. The intestinal flora is considered to be the second human genome, and its composition and function are closely related to the steady state of human health. At present, the research on the intestinal flora and many chronic diseases has gradually deepened, and many studies have reported various associations between the intestinal flora and T2DM in animals and humans (David et al., 2014; Everard et al., 2013). Intestinal flora disorders seem to affect the host's metabolism through the utilization of nutrients and the production of metabolites and promote susceptibility to metabolic disorders, such as insulin resistance and metabolic syndrome (Cani et al., 2007; Petruzzelli & Moschetta, 2010), in the high‐risk population of type 2 diabetes. These two factors are common in people at high risk of T2DM. There are significant differences in the composition of adult intestinal flora between diabetic patients and the control group. The difference in the composition of intestinal flora may affect the triggering factors of the occurrence and development of T2DM and its complications, such as energy extraction from food intake, mucosal immunity, and systemic inflammation (Bäckhed et al., 2004; Cani & Delzenne, 2007; Gravitz, 2012).

Among the intestinal flora, bifidobacteria and lactobacilli are considered to create a favorable intestinal environment and have significant benefits for healthy intestinal microbiota (Hill et al., 2014), thus becoming recognized as intestinal probiotics. The utilization of probiotics has garnered recognition as one of the most prevalent strategies for orchestrating alterations in the composition of the intestinal microbiota, potentially contributing to the mitigation or postponement of diabetes onset (Bordalo Tonucci et al., 2017). This study explored the effects of different prebiotics on intestinal probiotics in newly diagnosed diabetic patients based on these two probiotics.

Prebiotics are defined as indigestible food components, which can positively affect the composition and function of the intestinal microbiota by selectively stimulating the growth of bacteria present in the colon, thereby benefiting the health of the host (Schrezenmeir & de Vrese, 2001). As an illustration, milk contains an abundance of glycans that can function as prebiotics. The milk glycome encompasses free glycans, glycolipids, as well as N‐ and O‐glycosylated proteins (Kaplan et al., 2022). The glycan constituents liberated from milk glycoproteins exhibit the ability to selectively stimulate the proliferation of infant‐associated bifidobacteria, a pivotal gut microorganism for infants (Karav et al., 2016). In comparison to fully developed milk, bovine colostrum furnishes initial supplies of essential nutrients to neonates (Kaplan et al., 2021). N‐glycans originating from bovine colostrum hold promise as a robust reservoir of prebiotic substrates, with specific constituents potentially yielding anti‐infective effects, substantiated by recent findings demonstrating the anti‐infective properties of bovine colostrum‐sourced oligosaccharides against a notably invasive strain of Campylobacter jejuni (Arslan et al., 2021). Human milk oligosaccharides (HMOs), intricate and multifunctional free glycans present in both human and bovine milk, have the potential to invigorate the proliferation of beneficial microorganisms within the human intestinal tract by virtue of their prebiotic attributes. These compounds may possess substantial promise in the context of COVID‐19 infection treatments (Kaplan et al., 2022; Smith et al., 2013). The prebiotics undergo fermentation by the intestinal flora, yielding short‐chain fatty acids (SCFAs), which confer benefits to intestinal homeostasis and immunomodulation. Since different prebiotics produces different amounts and compositions of short‐chain fatty acids and gases after the fermentation of the microbiota, to prevent or treat certain inflammatory diseases, it is necessary to give priority to administration based on the metabolism of different prebiotic fibers in the colon (Tsai et al., 2019). The change in the intestinal flora of diabetic patients has been paid more and more attention by academic studies, but there is no targeted beneficial prebiotics for diabetic patients.

Multiple studies have demonstrated the potential impact of medications, such as metformin, on the composition of the intestinal microbiota (McCreight et al., 2016; Vallianou et al., 2019). However, existing research regarding the effects of prebiotics on diabetic patients has not yet definitively accounted for the influence of hypoglycemic drugs. To mitigate the confounding effects of medications on the intestinal microbiota, this study selected and enrolled newly diagnosed, untreated diabetic patients as the case group and healthy individuals as the control group. Sixteen distinct media, each containing various prebiotics, were chosen for experimentation. In vitro cultures of stool samples were performed to ascertain alterations in the degradation rates of beneficial bacteria and prebiotics. The objective was to investigate the impact of commonly employed prebiotics on the intestinal microbiota of newly diagnosed diabetic patients. These findings could potentially aid such patients in selecting targeted prebiotics tailored to their needs in the future. The study design encompassed in vitro cultivation of stool samples in 16 different media featuring diverse prebiotics. This approach enabled the assessment of changes in probiotic populations and prebiotic degradation rates. The overarching aim of our investigation is to elucidate the current effects of prevalent prebiotics on the intestinal microbiota of newly diagnosed diabetic patients, and thereby offer guidance for the selection of appropriate, targeted prebiotics in their future care.

## 2 METHODS

### 2.1 Participant characteristics

Individuals with abnormal blood sugar levels and those who underwent a medical examination at Huaxi Hospital between December 1, 2018, and December 31, 2018, were encompassed in this study. The eligibility criteria were as follows:

#### 2.1.1 Newly diagnosed diabetes group (case group)

Patients with untreated DM who were newly diagnosed within 1 year. According to the current international general diagnostic criteria and classification criteria for DM, thus fasting blood glucose ≥7.0 mmol/L or/and 2‐h postprandial blood glucose ≥11.1 mmol/L (World Health Organization, 1999), newly diagnosed patients with untreated DM were included.

#### 2.1.2 Healthy people (control group)

Included healthy people who took the physical examination in Huaxi Hospital. Health controls without history of diabetes mellitus were recruited from health checkup examinations and matched to patients by frequency according to age (±5 years) and gender.

#### 2.1.3 Exclusion criteria

Patients who had taken antibiotics within the past 2 weeks and intestinal probiotics‐related medications or kits within the past month, those who tested positive for fecal occult blood, individuals with severe liver or kidney impairment, and patients with diagnosed tumor conditions were all subjected to stringent screening and subsequently excluded. Following this meticulous screening process, a total of 10 cases were ultimately included in the case group, while another 10 cases were incorporated into the control group. Comprehensive clinical information, encompassing age, gender, waist and hip circumferences, smoking and alcohol consumption history, medical history, among others, was meticulously recorded. Additionally, serological data, including blood biochemistry, fasting and 2‐h plasma glucose measurements, fasting and 2‐h insulin levels, as well as glycated hemoglobin levels, were systematically documented. Patients were provided with dedicated containers to collect adequate stool samples. Blood biochemical indicators were analyzed using a Roche automatic biochemical analyzer and its corresponding kits (Roche). Glucose levels during fasting and 2‐h plasma glucose assessments were quantified via glucose oxidase assay (Roche), while fasting and 2‐h insulin levels were measured using a solid‐phase enzyme‐linked immunosorbent assay (Roche). Glycated hemoglobin was quantified using liquid chromatography (Roche). Prior to participation, the subjects were provided with comprehensive information about the study's objectives and procedures, and their informed consent was obtained in adherence to the “Declaration of Helsinki” as endorsed by the World Medical Association. Furthermore, the study was approved by the Ethics Committee of Huaxi Hospital (2018 No. 286).

### 2.2 In vitro fermentation

Under physiological conditions, fresh stool samples from the case group and the control group were respectively inoculated in nutrient media containing different prebiotics. The specific handling process is as follows:

An automatic stool homogenizer (China HaloBiotechnology Co.) was used to add 8 mL of 0.1 M anaerobic phosphate‐buffered saline (pH 7.0) to 0.8 ± 0.02 g of stool sample in a clean stool sample box to produce a 10% (w/v) fecal suspension. An aliquot of 0.5 mL of fecal suspension was then inoculated into 5 mL of culture medium, and anaerobic fermentation was carried out at 37°C. After 24 h of fermentation, the air pressure difference of the culture flask was measured. Subsequently, the medium was stored in a −30°C refrigerator for further metabolic analysis and DNA extraction.

### 2.3 Prebiotic selection

In this study, 16 prebiotics were selected as variables. The selection criteria are the prebiotics that is more common on the market and mentioned in the references that may affect the intestinal flora of diabetic patients (Kamada et al., 2013), mainly including the following 16 prebiotics: Lactulose (LAU, L), Raffinose (RAF, R), Fructo‐oligosaccharides (FOS, F), galacto‐oligosaccharides (GOS, G), Isomalto‐oligosaccharides (IMO, I), Mannan‐oligosaccharides (MOS, M), Xylo‐oligosaccharides (XOS, X), Inulin (INU, U), Soluble starch (STA, S), Mannite (MAI, A), Xylitol (XYI, B), Mucin (MUC, C), Sodium carboxymethyl starch (CS2, D), Glucose (GLU, E), YCFA+ Choline + L‐carnitine (YDZ), Soluble starch + Choline + L‐carnitine (SDZ). The above prebiotics were all added to the intestinal flora basal medium (YCFA, Y). The concentration of all prebiotics was 8 mg/mL per 100 mL basal medium +0.8 g prebiotics.

### 2.4 Intestinal probiotics testing

The CFX96 TM real‐time fluorescent quantitative PCR detection system (Bio‐Rad) was used to detect the number of *Lactobacillus* and *Bifidobacterium* in feces and culture media.

### 2.5 Detection of prebiotic degradation rate

Thin‐layer chromatography (TLC) was used to detect the degradation rate of oligosaccharides. The density of oligosaccharide spots in the TLC image was quantified using Quantity One software (Bio‐Rad). The consumption of oligosaccharides by bacteria was calculated by measuring the concentration difference between the culture medium with and without inoculation of stool. The degradation rate of oligosaccharides was the percentage of the amount consumed in its culture medium to its original amount.

### 2.6 Statistical methods

For statistical analysis, we used the SPSS Statistical Package (version 22.0, SPSS Inc.). Continuous variables conforming to normal distribution were expressed as mean ± standard deviation. A two‐tailed *t*‐test was used to compare normally distributed continuous variables between groups, and the correlation between prebiotics and beneficial bacteria increments was analyzed by linear regression. All *p* values are statistically significant when *p* < .05. Bonferroni corrections were applied.

## 3 RESULTS

### 3.1 Participant baseline data

A total of 20 participants were included in this study, including 10 cases in the case group and 10 cases in the control group. As shown in Table 1, there is no statistical difference in baseline data such as age and gender. The fasting blood glucose level and glycosylated hemoglobin of the case group were higher than those of the control group, and the difference was statistically significant (Table 1, *p* < .05).

**TABLE 1:** Baseline data of newly diagnosed diabetic patients and control group.

	Control group (*N* = 10)	Case group (*N* = 10)	*p* Value
Age	35.70 ± 5.92	43.30 ± 11.20	.112
Female (%)	30.0	40.0	1.000
Waistline	78.60 ± 3.53	83.0 ± 2.40	.140
Hipline	90.70 ± 2.50	93.8 ± 2.04	1.000
BMI	24.42 ± 1.61	27.48 ± 1.61	.961
Number of smokers (%)	60.0	70.0	1.000
ALB	52.01 ± 1.74	50.13 ± 2.02	.777
ALT	24.20 ± 9.11	36.80 ± 19.05	.141
AST	19.11 ± 7.45	23.49 ± 9.86	.218
CREA	46.79 ± 17.88	55.79 ± 27.35	.078
eGFR	109.33 ± 10.77	103.25 ± 17.31	.143
FBG	5.26 ± 0.38	7.34 ± 2.11	.005
2 h‐PG	–	13.20 ± 2.32	–
FINS	8.15 ± 1.35	8.68 ± 1.61	.434
2 h‐Ins	–	86.45 ± 34.46	–
HOMA‐IR	1.90 ± 30.31	2.73 ± 0.47	.459
HbA1c	5.36 ± 0.41	7.25 ± 1.11	.037*

Abbreviations: 2 h‐Ins, 2 h Postprandial insulin; 2 h‐PG, 2 h Postprandial blood glucose; ALB, Serum albumin; ALT, Alanine transaminase; AST, Aspartate aminotransferase; CREA, Serum creatinine; eGFR, Glomerular filtration rate; FBG, Fasting blood glucose; FINS, Fasting insulin; HbA1c, Glycosylated Hemoglobin; HOMA‐IR, Homeostasis model assessment of insulin resistance.

* *p* < .05.

### 3.2 The number of *Lactobacillus* and *Bifidobacterium* increased significantly after 24 h of culture

**FIGURE 1:**
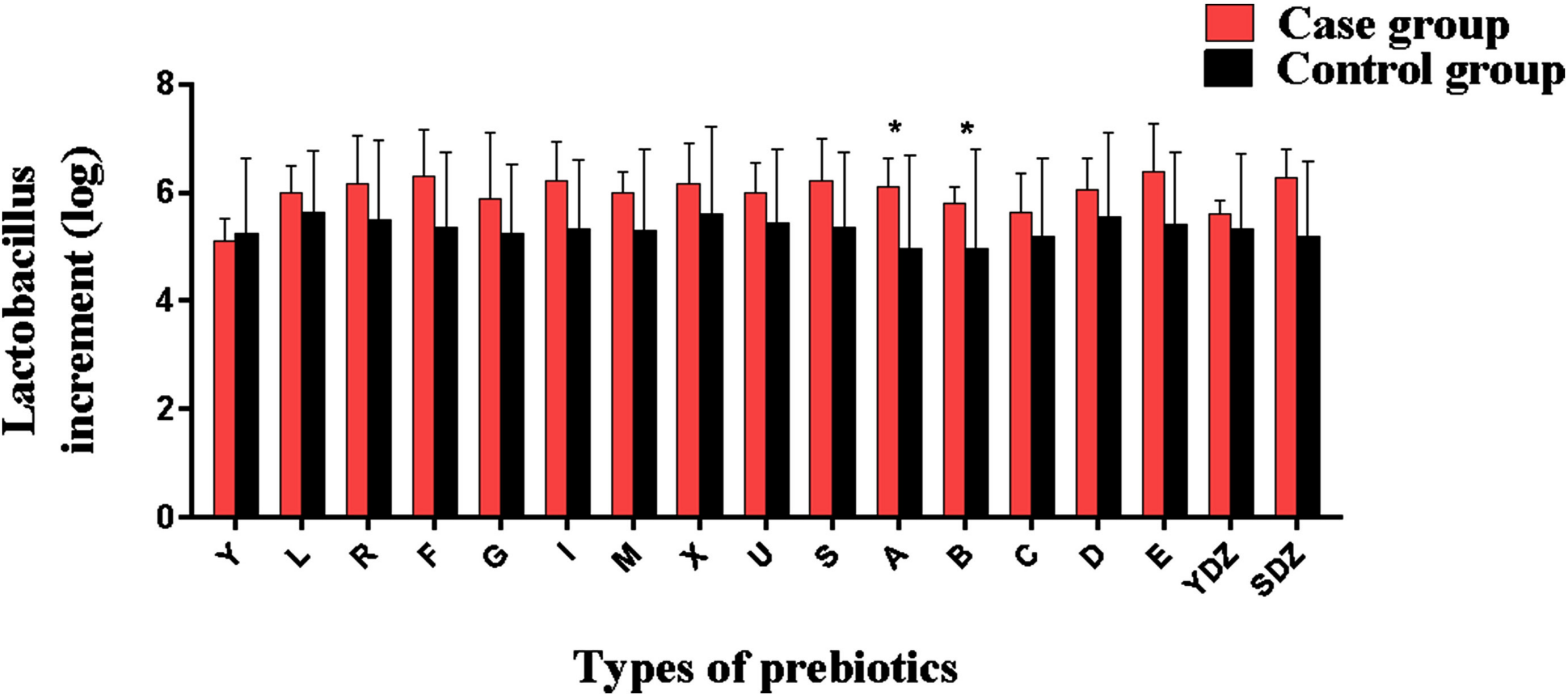
The increase in *Lactobacillus* in different prebiotic media. A, Mannite; B, Xylitol; C, Mucin; D, Sodium carboxymethyl starch; E, Glucose; F, Fructo‐oligos; G, Galacto‐oligosaccharides; I, Isomalto‐oligosaccharide; L, Lactulose; M, Mannan‐oligosaccharide; R, Raffinose; S, Soluble starch; SDZ, Soluble starch+ Choline+L‐carnitine; U, Inulin; X, Xylo‐oligosaccharides; YDZ, Y + Choline+L‐carnitine.*p < .05.

**FIGURE 2:**
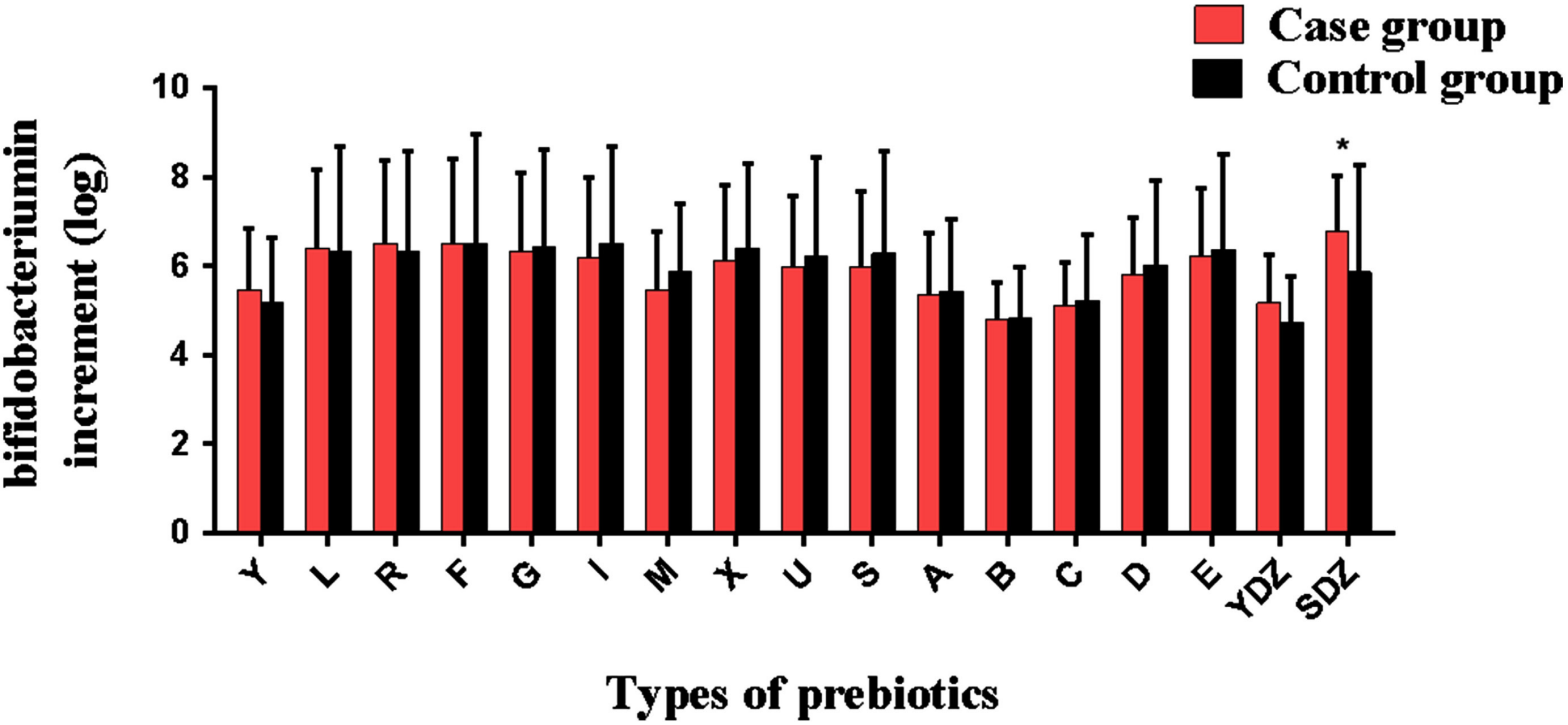
The increase in *Bifidobacterium* in media containing different prebiotics. A, Mannite; B, Xylitol; C, Mucin; D, Sodium carboxymethyl starch; E, Glucose; F, Fructo‐oligos; G, Galacto‐oligosaccharides; I, Isomalto‐oligosaccharide; L, Lactulose; M, Mannan‐oligosaccharide; R, Raffinose; S, Soluble starch; SDZ, Soluble starch+ Choline+L‐carnitine; U, Inulin; X, Xylo‐oligosaccharides; YDZ, Y + Choline+L‐carnitine.*p < .05.

The stool samples of the two groups of patients were fermented in the culture medium for 24 h, and the increase in *Lactobacillus* and *Bifidobacterium* was observed. As shown in Figure 1, in the medium containing xylitol and mannitol, the increase in *Lactobacillus* in the case group was more than that in the control group, and the difference was statistically significant (*p* < .05). In the medium containing soluble starch + choline + L‐carnitine, the increase in *Bifidobacterium* in the case group was greater than that in the control group, and the difference was statistically significant (Figure 2, *p* < .05).

### 3.3 Degradation rate of oligosaccharide prebiotics

**FIGURE 3:**
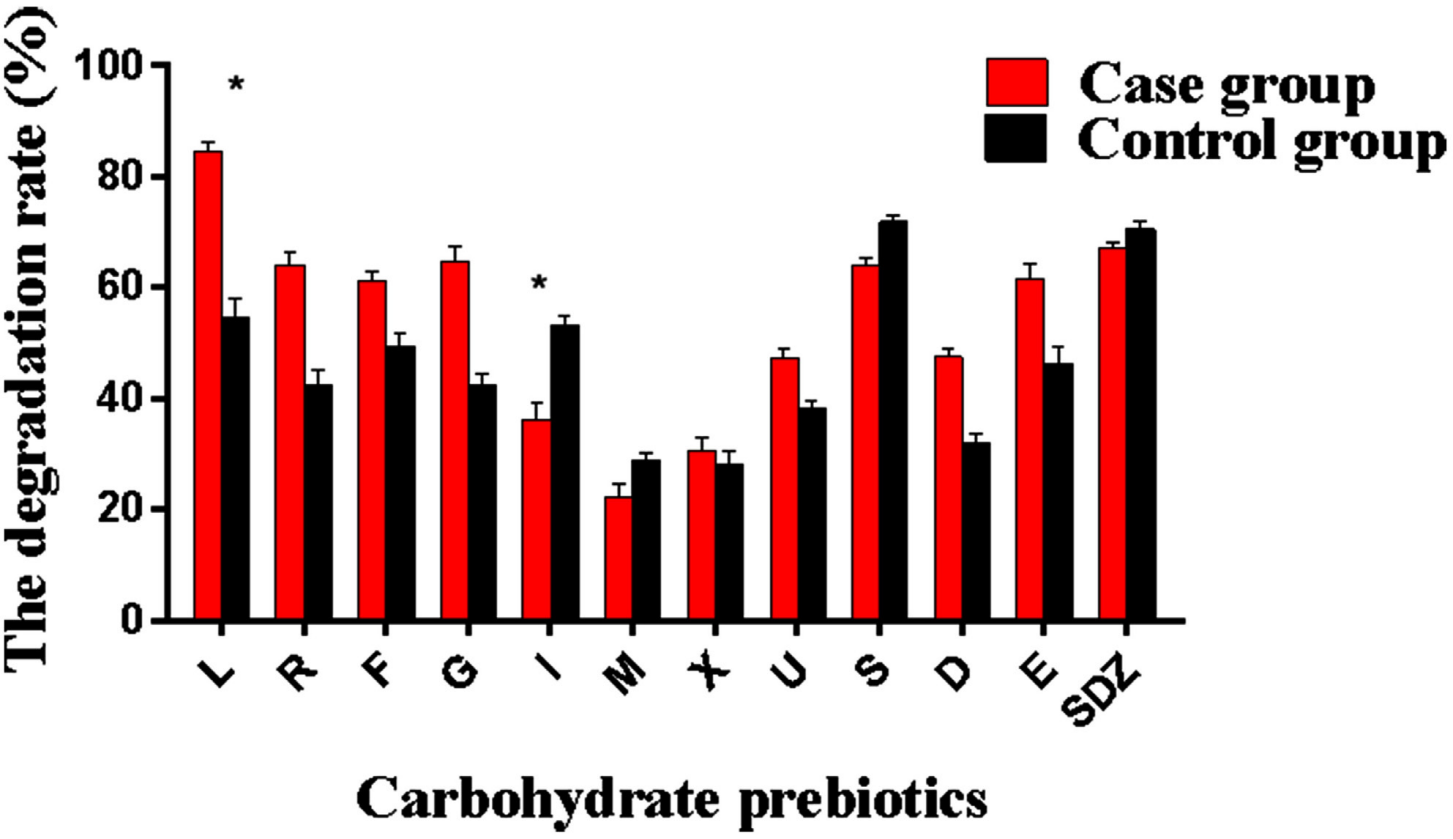
Degradation rate of carbohydrate prebiotics. D, Sodium carboxymethyl starch; E, Glucose; F, Fructo‐oligos; G, Galacto‐oligosaccharides; I, Isomalto‐oligosaccharide; L, Lactulose; M, Mannan‐oligosaccharide; R‐Raffinose; S, Soluble starch; U, Inulin; X, Xylo‐oligosaccharides. *p < .05.

After fermenting the stool samples of the two groups of participants for 24 h, the degradation rate of oligosaccharide prebiotics was observed. As shown in Figure 3, in the medium containing lactulose (L) and isomaltooligosaccharides (I), the difference in sugar degradation rate between the case group and the control group was statistically significant, and the case group had a higher degradation rate of lactulose, while the degradation rate of isomaltooligosaccharides is lower than the control group.

### 3.4 Correlation between the degradation rate of prebiotics and probiotics

This study further analyzed the correlation between the degradation rate of prebiotics and the increase in probiotics. As shown in Table 2, in the medium containing fructooligosaccharides, galactooligosaccharides, isomaltooligosaccharides, and inulin, the degradation rate of prebiotics is positively correlated with the increase in *Bifidobacterium*, and the correlation is statistically significant. There is no obvious statistical correlation between the increase in *Lactobacillus* and the degradation rate of prebiotics (Table 2).

**TABLE 2:** Correlation between the degradation rate of prebiotics and probiotics.

Prebiotics	*Bifidobacterium*		*Lactobacillus*	
	Correlation coefficient *R*	*p* Value	Correlation coefficient *R*	*p* Value
L	.418	.067	−.15	.527
R	.290	.216	−.28	.232
F	.461	.041*	−.127	.594
G	.453	.045*	.044	.854
I	.508	.022*	−.258	.272
M	.132	.578	.148	.533
X	.407	.075	.258	.272
U	.650	.002*	−.239	.310
S	.177	.456	−.163	.491
D	−.123	.605	−.106	.657
E	.226	.337	−.102	.667
SDZ	−.138	.561	−.078	.745

Abbreviations: A, Mannite; B, Xylitol; C, Mucin; D, Sodium carboxymethyl starch; E, Glucose; F, Fructo‐oligos; G, Galacto‐oligosaccharides; I, Isomalto‐oligosaccharide; L, Lactulose; M, Mannan‐oligosaccharide; R‐Raffinose; S, Soluble starch; SDZ, Soluble starch+ Choline+L‐carnitine; U, Inulin; X, Xylo‐oligosaccharides; YDZ, Y + Choline+L‐carnitine.

* *p* < .05.

## 4 DISCUSSION

Prebiotics has been reported to improve insulin resistance and obesity through probiotics. However, the pre‐ and post‐relationship underlying these effects remain unclear. In this study, we investigated the prebiotics, such as xylitol and mannitol which can increase intestinal probiotics in newly diagnosed diabetic patients. We found the changes in the balance of intestinal flora in diabetic patients may be manifested in the degradation rate of certain types of prebiotics, and thus the adjustment of intestinal flora by prebiotics may become a new direction of research for the treatment of diabetes.

The human gut microbiota is composed of trillions of microorganisms from more than 1000 species (Barengolts, 2016). Due to the diversity of intestinal microbes and their metabolites and the high complexity of their functions, they have increasingly become the focus of research on chronic diseases, including cancer, inflammation, metabolic diseases, cardiovascular diseases, autoimmune diseases, neurological diseases, and mental diseases. T2DM is the result of complex gene–environment interactions. The intestinal flora is considered an environmental factor related to T2DM, which participates in a complex gene–environment interaction network and promotes the occurrence and development of diabetes (Sabatino et al., 2017). The main characteristics of the intestinal flora and metabolism of patients with T2DM are dysbacteriosis, chronic inflammation of the intestine, increased intestinal permeability, and changes in mucosal immune response, etc. (Cani, 2018; Razmpoosh et al., 2019). Probiotics and prebiotics are some of the most widely used methods to regulate intestinal flora. After effective prebiotics/probiotics are ingested, changes in the composition and activity of the intestinal flora may systematically regulate the gene expression patterns (transcriptomics) and metabolism (metabolomics) of many organs of the host. Probiotics can regulate the interaction between intestinal flora and the immune system. Prebiotics can promote the growth of certain bacterial species, stabilize the mucosal function, reduce the abundance of pathogenic bacteria through acidification of the intestinal lumen, produce antibacterial substances, overcome nutritional competition (Griffiths et al., 2004), and thus play a role in preventing or delaying the occurrence of diabetes (Steer et al., 2000). In the human intestinal tract, the intestinal flora is diverse in composition, large in number, and complex in function.

At present, there is few research to confirm the specific role of each intestinal bacteria, but a large number of studies have confirmed that *Lactobacillus* and *Bifidobacterium* are the main probiotics (Tsai et al., 2019). The study of Parnell and Reimer (2009) showed that compared with a placebo, oligofructose can reduce plasma glucose and insulin levels in obese patients. Dehghan et al. (2014) found that inulin rich in oligofructose can reduce fasting blood glucose and glycosylated hemoglobin in diabetic patients, as well as inflammation markers (IL‐6, TNF‐α) and lipopolysaccharide (LPS). Another study showed that in healthy subjects or patients with metabolic syndrome, consumption of resistant starch can increase insulin sensitivity, and in patients with T2DM, consumption of resistant starch can reduce postprandial blood sugar or insulin (Bodinham et al., 2014). Therefore, this study focused on the detection and analysis of *Lactobacillus* and *Bifidobacterium* in the stool of the participants. *Lactobacillus* has many benefits to host health, including the regulation of blood glucose and lipid metabolism by regulating the expression of genes related to glucose and lipid metabolism (Yan et al., 2019). *Lactobacillus* improves the intestinal epithelial barrier function, improves oxidative stress, reduces IL‐8, TNF‐α, IL‐1β, and other inflammatory cytokines in the liver and colon tissues, which prevents liver and colon tissue damage to a certain extent (Yan et al., 2019). Like *Lactobacillus*, *Bifidobacterium* can inhibit harmful bacteria, improve the barrier function of the gastrointestinal tract, and inhibit pro‐inflammatory cytokines. *Bifidobacterium* can also change the function of dendritic cells to regulate intestinal immune homeostasis (Wang et al., 2017). Besides, *Bifidobacterium* can metabolize bacteria, and cross‐feeding can increase the proportion of beneficial bacteria in the intestinal flora. Although the effects of probiotics are remarkable, prebiotics has significant advantages, including greater resistance to digestive disorders, lower cost, lower risk, and relatively easy acceptance by the food industry. Therefore, in recent years, the trend of the application of prebiotics has gradually exceeded the trend of probiotics (Hoarau et al., 2006).

This study included prebiotics that is currently widely used and recognized to have a certain impact on the intestinal flora. Through experiments, it was found that in the medium containing xylitol and mannitol, the increase in *Lactobacillus* was higher in T2DM patients, while in the medium containing soluble starch + choline + L‐carnitine, the increase in *Bifidobacterium* was higher. This is consistent with the conclusions of previous animal studies (Hoarau et al., 2006): prebiotic treatment can increase the number of probiotics (mainly *Lactobacillus* and *Bifidobacterium*) in the intestinal flora and have a beneficial effect on the disease. Xylitol has been reported as a potential anti‐diabetic sweetener in some recent studies. It can improve the morphology of pancreatic islets and reduce blood glucose, but the anti‐diabetic mechanism is still unclear (Kishore et al., 2012; Rahman & Islam, 2014). Studies have shown that xylitol can inhibit gastric emptying and increase satiety by promoting the release of glucagon‐like peptide‐1 and cholecystokinin. It can also effectively reduce the accumulation of visceral fat, which is beneficial for weight control and reducing insulin resistance (Amo et al., 2011; Wölnerhanssen et al., 2016). While mannitol is mainly used as a dehydrating agent in the treatment of intracranial hypertension, few studies have studied its effect on intestinal flora. Our research suggests that xylitol and mannitol may improve the intestinal flora of diabetic patients, increase probiotics, and play a role in hypoglycemia, but their specific mechanisms still need to be further studied. Current studies believe that the degradation rate of various prebiotics in the colon has a certain range and that too high or too low degradation rates indicate changes in the steady state of the intestinal flora (Luoto et al., 2010; Lyssenko et al., 2008). Our research suggests that the intestinal flora of diabetic patients have a higher degradation rate of lactulose, but a lower degradation rate of isomaltooligosaccharides. It is worth noting that only the degradation of some prebiotics we used (saccharide prebiotics) was analyzed in this study, and the specific mechanism of prebiotics' degradation promoting the growth of probiotics was not explored, that is what we lack and needs further research. But our research at least makes it clear that the homeostasis of the intestinal flora of diabetic patients has indeed changed and the change can be manifested by the degradation rate of certain types of prebiotics, which may be used to predict early diabetes.

In various animal studies, prebiotics has been shown to improve glucose metabolism (Everard et al., 2011; Kok et al., 1998). However, this is inconsistent with the results of research on humans (Kim et al., 2018). This may be because the efficacy of human prebiotics and probiotics is much more complicated than animal experiments. Many factors such as dietary characteristics, drug use, and body mass index, may affect the intestinal microbiota of the subject. At the same time, glucose metabolism, insulin secretion, and other intestinal hormones such as incretin can also affect the intestinal microbiota (Hooper & Gordon, 2001). Therefore, future studies should consider these factors to better understand the metabolic effects of prebiotics/probiotics on diabetic people and the main mechanisms involved in this complex relationship.

## 5 CONCLUSION

Our study indicates that prebiotics, including xylitol and mannitol, have the potential to enhance the levels of intestinal probiotics in newly diagnosed diabetic patients. This augmentation could potentially contribute to a certain level of restraint on the onset and progression of diabetes. The alterations observed in the equilibrium of intestinal flora among diabetic patients may be linked to variations in the degradation kinetics of specific prebiotic types. The potential for modulating intestinal microbiota via prebiotic interventions suggests a novel avenue for exploring diabetes management strategies.

## AUTHOR CONTRIBUTIONS

**Yu Zhang:** Writing – original draft (equal). **Lidan Yang:** Data curation (equal). **Yitian Wu:** Data curation (equal). **He He:** Data curation (equal). **Yuping Zeng:** Data curation (equal). **Zhenmei An:** Data curation (equal). **Weiguo Jia:** Writing – review and editing (equal).

## FUNDING INFORMATION

This work was supported by the Sichuan Science and Technology Department (grant number: 2018SZ0112), and the National Natural Science Foundation of China (grant number: 32071462).

## CONFLICT OF INTEREST STATEMENT

Yu Zhang, Lidan Yang, Yitian Wu, He He, Yuping Zeng, Zhenmei An, and Weiguo Jia declares that they have no conflict of interest.

## ETHICS STATEMENT

The present study was approved by the Ethics Committee of Huaxi Hospital. Informed consent was obtained from all patients.

## CONSENT FOR PUBLICATION

Not applicable.

## Data Availability

The datasets used and/or analyzed during the current study are available from the corresponding author on reasonable request.
